# Retinal Fractal Dimension Is a Potential Biomarker for Systemic Health—Evidence From a Mixed-Age, Primary-Care Population

**DOI:** 10.1167/tvst.13.4.19

**Published:** 2024-04-12

**Authors:** Justin Engelmann, Stephanie Kearney, Alice McTrusty, Greta McKinlay, Miguel O. Bernabeu, Niall Strang

**Affiliations:** 1 Centre for Medical Informatics, Usher Institute, University of Edinburgh, Edinburgh, UK; 2 School of Informatics, University of Edinburgh, Edinburgh, UK; 3 Department of Vision Sciences, Glasgow Caledonian University, Glasgow, UK; 4 Centre for Clinical Brain Sciences, University of Edinburgh, Edinburgh, UK; 5 The Bayes Centre, University of Edinburgh, Edinburgh, UK

**Keywords:** fractal dimension, oculomics, retinal image analysis

## Abstract

**Purpose:**

To investigate whether fractal dimension (FD), a retinal trait relating to vascular complexity and a potential “oculomics” biomarker for systemic disease, is applicable to a mixed-age, primary-care population.

**Methods:**

We used cross-sectional data (96 individuals; 183 eyes; ages 18–81 years) from a university-based optometry clinic in Glasgow, Scotland, to study the association between FD and systemic health. We computed FD from color fundus images using Deep Approximation of Retinal Traits (DART), an artificial intelligence–based method designed to be more robust to poor image quality.

**Results:**

Despite DART being designed to be more robust, a significant association (*P* < 0.001) between image quality and FD remained. Consistent with previous literature, age was associated with lower FD (*P* < 0.001 univariate and when adjusting for image quality). However, FD variance was higher in older patients, and some patients over 60 had FD comparable to those of patients in their 20s. Prevalent systemic conditions were significantly (*P* = 0.037) associated with lower FD when adjusting for image quality and age.

**Conclusions:**

Our work suggests that FD as a biomarker for systemic health extends to mixed-age, primary-care populations. FD decreases with age but might not substantially decrease in everyone. This should be further investigated using longitudinal data. Finally, image quality was associated with FD, but it is unclear whether this finding is measurement error caused by image quality or confounded by age and health. Future work should investigate this to clarify whether adjusting for image quality is appropriate.

**Translational Relevance:**

FD could potentially be used in regular screening settings, but questions around image quality remain.

## Introduction

Retinal color fundus imaging rapidly and non-invasively captures a detailed picture of the retinal vasculature while being widely available and low cost. Thus, retinal image–derived traits are being investigated as biomarkers for systemic health, a field also known as “oculomics.”^1^^–^^3^ Fractal dimension (FD), a retinal trait relating to the complexity of the retinal vasculature, has emerged as a particularly promising candidate that is associated with cardiovascular^4^^,^^5^ and neurovascular^6^^,^^7^ disease. FD is calculated from vessel segmentations and captures how complex the branching structure of the blood vessels is. A lower FD indicates a less complex vasculature, which might be an indicator for poorer retinal vessel health. This in turn could indicate poorer vessel health systemically, and, indeed, individuals with lower FD are at higher risk of cardiovascular disease,^4^ for example. Thus, retinal FD derived from color fundus images could serve as a potential biomarker for systemic health in research and clinical practice that could identify individuals at high risk who could then be examined in more detail.

However, research to date has focused on cohort studies such as the UK Biobank or data from secondary care settings such as AlzEye.^8^^,^^9^ These are generally older and not representative of the broader population. Mean age is 57.8 ± 8.6 years in the UK Biobank^10^ and 68.4 ± 13.9 years in AlzEye. In addition to the participants being older, the UK Biobank has more female participants and fewer who are socioeconomically deprived than the general UK population,^11^ and AlzEye participation was predicated on hospital attendance. This leaves open the question of whether FD as a biomarker for systemic health is applicable to mixed-age, primary-care populations—populations that span from young adults all the way to advanced age who have been assessed in a standard primary-care setting.

Previous work looking at retinal vascular traits and systemic health is also limited by the exclusion of large amounts of data due to image quality, on the order of 20% to 45% even for datasets such as the UK Biobank that are specifically collected for research.^2^^,^^4^^,^^5^ Even rejecting one in five images would drastically limit the utility of FD in clinical practice. Furthermore, older, less healthy, male, and non-White subjects are at higher risk of being excluded due to image quality.^12^ Thus, quality-based exclusions introduce selection bias that could exacerbate existing disparities in healthcare research.

Recently, a novel artificial intelligence–based method for computing FD has been proposed: Deep Approximation of Retinal Traits (DART).^13^ Traditional methods such as VAMPIRE^14^ calculate FD from binary segmentations of the blood vessels, and even small imperfections in those segmentations can affect the calculations, resulting in a high bar for minimum image quality. DART uses a deep learning model that was trained to output the same FD as VAMPIRE but during training received original high-quality images and degraded versions of those, such as with altered brightness or contrast to simulate lighting issues, blur, or simulated artifacts. The model was tasked to output the number VAMPIRE gave for the original high-quality image and thus learned to ignore variations in image quality and take into account all available information about vessel structure to estimate FD. Intuitively, even if only part of the vessels is highly visible in a fundus image, the image still contains substantial information about the vasculature. DART can leverage this information, whereas traditional approaches are not robust to such cases. DART has shown very high internal validity on held-out UK Biobank images, matching VAMPIRE with a Pearson correlation of 0.9572 when DART received the original image and a correlation of 0.8817 when DART received severely degraded images instead (both *P* < 0.0001).

In this work, we investigated the potential of using FD as a potential biomarker for systemic health in clinical practice by studying a mixed-age, primary-care population. To avoid introducing bias and to evaluate the robustness of FD under challenging, real-world conditions, we made no image quality exclusions.

## Methods

### Glasgow Caledonian University Cohort

Clinical records from eye examination appointments that took place between 2017 and 2022 at the Vision Centre at Glasgow Caledonian University (GCU), Glasgow, Scotland, United Kingdom, were analyzed. As part of the eye examinations, a record card was completed, and color fundus images were captured for each patient. The study was undertaken in accordance with the tenets of the Declaration of Helsinki. Ethical approval was obtained from the GCU School of Health Sciences Ethical Committee prior to the commencement of data collection (HLS/LS/A22/003). Participants provided written consent to have their anonymized clinical records used for research purposes. Retinal images were obtained using swept-source optical computed tomography (DRI OCT Triton Plus; Topcon, Tokyo, Japan).

The record cards were either digitized if they were in physical paper form or extracted from the patient management system. All of the images were available digitally on the device and exported in JPG format at a resolution of 2000 × 1312 pixels or greater and linked to the record cards. The following information was then collated in a consistent format: age at visit, sex, and information about the general health status from the “history and symptoms” field in the record card.

We had access to 183 images linked to 96 records belonging to 58 female and 38 male patients. In nine cases, only a single image was available on the device, presumably because only one eye was imaged during the examination. For consistency, we used the most recent visit for each individual, and all available images were included in our analysis. The data extraction process was labor intensive; thus, we had access to only one visit per patient. During data extraction, we prioritized extracting data for as many patients as possible at the cost of not extracting longitudinal data. The median age at visit was 61.80 years (interquartile range [IQR], 33.20) with the youngest and oldest patients being 18 and 81 years, respectively. Notably, 22 patients were under the age of 30, an age group that is not available in UK Biobank or AlzEye where the youngest subjects are 37^10^ and 40,^9^ respectively.

### Systemic Health Information

We used information about systemic health from the “history and symptoms” field of the record cards to analyze the relationship between systemic health and FD. This information was recorded in the context of an optometric examination and thus was generally coarse grained with varying levels of detail, primarily consisting of very short descriptions and commonly used clinical abbreviations (e.g., “Good, no problems,” “diabetes”, “HBP”). We stratified individuals into two groups based on this information: those described as having any systemic health condition (i.e., any non-ocular health condition) and those with no mention of any such condition. Table 1 gives an overview of the two groups.

**Table 1.** Population Characteristics Stratified by Systemic Health Status

	All	Prevalent Systemic Condition	No Systemic Condition
*n*	96	46	50
Female, %	60.4	52.2	68.0
Age (y), median ± IQR	61.80 ± 33.20	62.79 ± 9.60	55.28 ± 39.51

Although the level of detail for the systemic health information is limited at times, this approach should have high positive predictive value; that is, individuals in the “prevalent systemic conditions” group would be very likely to actually have systemic conditions. Sensitivity, on the other hand, would be imperfect. Prevalent conditions might not have been mentioned by the patient, might not have been deemed sufficiently relevant to record, or might have been undiagnosed at the time of visit. This should lead to lower apparent effect sizes. Another limitation is that we do not have information about the severity or duration of the conditions. This should likewise lead to lower apparent effect sizes than if we could account for severity. Thus, we expect that the limitations of this variable biased our analysis to be more conservative, and any apparent differences between the two groups are likely to be true differences but with underestimated effect sizes; in other words, the risk of a type 1 error was low and that of a type 2 error was high.

### Computing FD and Image Quality Annotation

To compute the FD of the images, we used DART,^13^ which is based on the multifractal FD of VAMPIRE.^2^^,^^14^^,^^15^ All images could be successfully processed in less than a minute on a consumer-grade, desktop central processing unit (CPU), and no images were excluded from analysis. In addition to computing FD, we manually annotated retinal image quality on a four-level ordinal scale (very good, good, poor, or very poor). The images were annotated by an experienced research optometrist with 10 years of clinical experience (S.K.). Previous work by Laurik-Feuerstein et al.^16^ found good intergrader agreement for a similar four-level ordinal taxonomy for color fundus images. Poor-quality fundus images are common even in research datasets such as the UK Biobank, where researchers typically exclude 20% to 40% of the available images. In our dataset, 34.6% of the images were rated as very good, 33.9% as good, 28.1% as poor, and 3.4% as very poor. The proportion of our images rated as poor or very poor (31.5%) is comparable to the 20% to 45% of images that are typically excluded in the UK Biobank.^2^^,^^4^^,^^5^ Examples for each of the four quality levels are shown in Supplementary Figure S1.

### Statistical Analysis

We first analyzed the FD values computed by DART to see whether they showed the expected association with age and whether there was an association with image quality or sex that we needed to adjust for. We then used a linear mixed-effects model at the eye level with FD as the dependent variable and a random intercept per patient to adjust for the two eyes of an individual not being independent. The data was analyzed using the statsmodels^17^ package (version 0.13.5) in Python 3.9.13. We used a threshold of *P* < 0.05 for statistical significance throughout.

For retinal traits relating to vessel caliber, differences in magnification due to refractive error (RE) can change the apparent size of vessels.^18^^,^^19^ We expect that for FD this is not the case, as FD relates to the branching structure of the vessels and especially because VAMPIRE, on which DART is based, uses skeletonized vessel maps and should thus be invariant to apparent caliber. However, we also empirically investigated this to ensure that our decision not to include RE did not affect our results.

## Results

### FD, Image Quality, Age, and Sex

The mean FD in our data was 1.4904, with a standard deviation (SD) of 0.0391. An increase in image quality issues by one level was associated with a decrease in FD by 0.026 (95% confidence interval [CI], 0.031–0.021) in absolute terms, or by 0.656 SD (95% CI, 0.788–0.524; *P* < 0.001). This suggests that, although DART is designed to be more robust to image quality issues, there might still be an effect that must be adjusted for.

**Figure 1.**
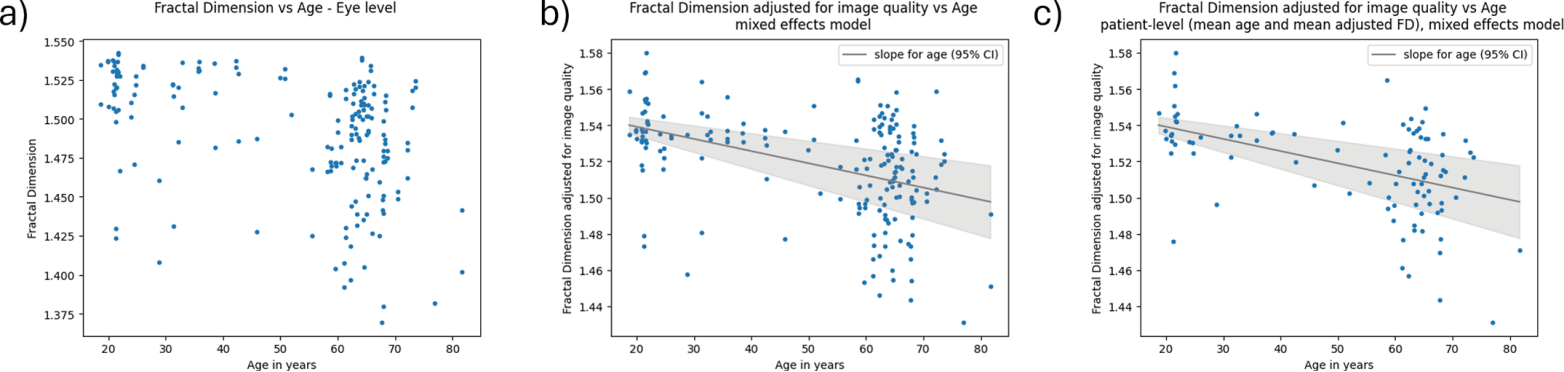
Association of retinal FD and age. (**a**) Raw data. (**b**) FD adjusted for image quality using a mixed-effects model with random intercepts for each patient at the eye-level (i.e., each point corresponds to one eye). (**c**) The same model as in part (**b**) but with values at the patient level (i.e., each point is one patient). For parts (**b**) and (**c**), the coefficient for age is −0.007 per decade of age (95% CI, −0.009 to −0.004; *P* < 0.001).

Figure 1 shows scatterplots of FD versus age for the raw data, as well as when FD was being adjusted for image quality in a mixed-effects model. Increasing age was associated with a significant decrease in FD. In a univariable model, an additional decade of age was associated with a decrease in FD by 0.232 SD (95% CI, 0.323–0.141; *P* < 0.001). When adjusting for image quality issues, this changed to a decrease by 0.172 SD per decade (95% CI, 0.235–0.109; *P* < 0.001), which is consistent in direction and magnitude with what has been reported in the literature.^20^ Furthermore, although the effect of age on FD did decrease after adjusting for image quality issues, the direction was the same and the magnitude comparable. This suggests that, although the effect of image quality is significant, it does not preclude discovering meaningful associations in our data.

In addition to the linear association between age and FD, we also observed that the FD variance was higher in older patients. Some patients over 60 had FD comparable to those of patients in their 20s (Fig. 1a). This suggests that patients could follow characteristically different trajectories, with only some seeing their FD decrease substantially as they age. The findings persisted when adjusting for image quality using the coefficients from the linear mixed-effects model (Fig. 1b) and when taking the mean of both eyes per patient (Fig. 1c). Sex showed no significant association with FD in a univariable model (*P* = 0.252), when adjusted for image quality (*P* = 0.156) and when adjusted for image quality and age (*P* = 0.377).

### FD and Systemic Disease

As our preliminary analysis suggested that age and image quality are significantly associated with FD, we adjusted our model for these variables. We fit the following mixed-effects model with a random intercept per patient: FD ∼ age + image quality + prevalent systemic conditions + (1|patient). Table 2 shows the resulting model. Prevalent systemic conditions were significantly associated with a decrease in FD by 0.246 SD (95% CI, −0.477 to −0.015; *P* = 0.037). In a univariable model, the decrease was 0.461 SD (95% CI, −0.833 to −0.088; *P* = 0.015) and was 0.361 SD (95% CI, −0.612 to −0.110; *P* = 0.005) when just adjusting for image quality only.

**Table 2.** Mixed-Effects Model on FD

Variable	β	95% CI	*P*
Intercept	39.711	39.369 to 40.054	0.000
Age (in decades)	−0.158	−0.221 to −0.095	0.000
Image quality (ordinal, higher is worse)	−0.631	−0.750 to −0.512	0.000
Prevalent systemic conditions	−0.246	−0.477 to −0.015	0.037
Random effect group variance	0.187	—	—

All coefficients are in standard deviation units of FD.

### FD and Refractive Error

We split eyes into three groups based on their RE: hyperopic (RE > 1.5; 30 eyes), myopic (RE < −1.5; 50 eyes), and emmetropic. When added to the model in Table 2, neither being hyperopic nor being myopic had a significant effect (*P* = 0.159 and *P* = 0.204, respectively), and prevalent systemic conditions had the same coefficient (β = −0.246) with a slightly reduced *P* value of 0.041, possibly due to residual cofounding between RE and age. When looking at RE adjusted only for age, there likewise were no significant associations with FD, with *P* values of 0.159 and 0.204 for being hyperopic and myopic, respectively. Thus, RE does not appear to have influenced the FD measurements in our dataset and would not have meaningfully affected our results if we had included it in our model.

## Discussion

### FD as a Biomarker for Systemic Health

We observed a significant association between prevalent systemic conditions and FD, even when controlling for age and image quality. This confirms previous findings that FD might be a biomarker for systemic health. This is further corroborated by the observation that FD had higher variance in older patients. Importantly, our data was collected during clinical practice in a primary-care setting and included patients from 18 to 81 years of age, and no images were excluded from analysis based on quality. Thus, our work suggests that FD can be used in an even more challenging setting that is closer to clinical reality than what previous work considered.

A major limitation is the coarseness of the information about prevalent systemic conditions and the lack of information about incident systemic conditions. However, as we argued in the Methods section, we expect that the variable for prevalent systemic conditions will have high positive predictive value; thus, the observed effect is likely to be a true effect and the effect size will be underestimated. Future work should further explore FD as a biomarker for systemic health in primary-care data, ideally with linkage to other medical records for more detailed information about systemic health.

### Should Image Quality be Adjusted for?

We adjusted for image quality due to its strong association with FD, which is commonly done in the literature.^4^ The underlying assumption is that image quality issues affect the computation of FD itself and thus must be adjusted for to recover retinal vascular complexity. This notion initially appears convincing, but upon reflection is potentially problematic.

Retinal vascular complexity is supposed to be a proxy for systemic health; however, age and poorer health have been found to be associated with not just vascular complexity but also image quality itself.^12^ Plausibly, frailer individuals could be more difficult to image (e.g., because of difficulty sitting still). Furthermore, age-related changes to the eye, such as miosis and cataract, also decrease expected image quality.

**Figure 2.**
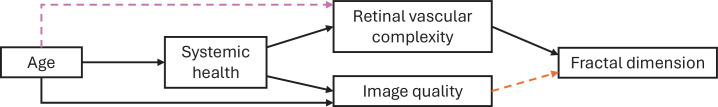
Simplified causal diagram for FD measurements. The *dashed orange arrow* indicates the undesirable potential effect of image quality on FD. The *dashed pink arrow* indicates a potential direct effect of age on retinal vascular complexity that could occur in healthy aging.

Figure 2 shows a simplified causal diagram for FD measurements, with a potential direct effect of age on retinal vascular complexity (dashed pink arrow). To our knowledge, this effect has not been conclusively established in the literature but could plausibly exist. The dashed orange arrow indicates a potential effect of image quality on FD, which is the reason why image quality is commonly adjusted for.

However, observe that, even if we had a fully robust method where this arrow would not be present, we would expect to observe a significant association between FD and image quality, confounded by systemic health. In that case, adjusting for image quality would be inappropriate and would bias the association between FD and systemic health toward the null. Even if our method is not perfectly robust and there is an effect of image quality on FD (i.e., poor-quality images lead not only to measurement noise but also to a systematic bias in FD), then controlling for image quality could likewise bias our analysis.

Thus, we argue that adjusting for image quality is potentially problematic and should be more critically considered in oculomics research. This issue further highlights the need for robust methods for computing retinal traits, as more robust methods would reduce the need to adjust for image quality in the first place.

Note that this issue cannot be side-stepped by only examining high-quality images and excluding the rest. As mentioned in the Introduction, these exclusions introduce selection bias and exacerbate inequalities in healthcare research,^12^ in addition to reducing sample sizes and statistical power. Future work should look at the repeatability and robustness to image quality of DART and traditional approaches to add empirical evidence to the question of whether adjusting for image quality can be avoided.

### Age and FD

Our finding that some older patients have FD similar to those of younger patients suggests that FD might not substantially decrease with age in all individuals. Likewise, the increased variance of FD in older patients suggests that individual trajectories might be quite heterogeneous. It also suggests that FD does not merely change with age, lending credence to the hypothesis that it captures meaningful biological changes and that it could provide information about systemic health beyond what age itself provides. However, our analysis here is cross-sectional and future work should investigate this in longitudinal data.

## Conclusions

In addition to the aforementioned limitation relating to the systemic health information, our work has several additional limitations. First, only a single quality annotator was used, although they were very experienced, and comparable quality taxonomies for color fundus imaging have good repeatability according to the literature. Future work ideally should use fully automated methods such as the recently proposed QuickQual^21^ that avoid introducing subjectivity and allow better comparison of quality annotations across different works. Second, although DART is more robust to image quality than traditional approaches, some images might still be too poor in quality. In the present dataset, even the “very bad” images had at least some visible vasculature that could provide sufficient information for a reasonable FD estimate (see Supplementary Fig. S1). Nevertheless, this issue should be further investigated in future work. Third, despite the promising results in the present work and literature at large, future work should also more closely investigate what specific vascular changes are captured by FD.

In summary, we found that prevalent systemic health conditions are associated with a significant decrease in FD in a mixed-age, primary-care population. Furthermore, although FD generally decreases with age, our data suggest that FD might not substantially decrease in everyone. This heterogeneity could be due to systemic health, further supporting FD as a potential biomarker for systemic health. Future work should study the relationship between FD and age in longitudinal data and clarify whether adjusting for image quality is appropriate.

## Supplementary Material

Supplement 1
