# Supplementary material for: Retinal Fractal Dimension Is a Potential Biomarker for Systemic Health—Evidence From a Mixed-Age, Primary-Care Population
Source: Transl Vis Sci Technol. 2024 Apr 12;13(4):19. doi: 10.1167/tvst.13.4.19 (PMC11019596; doi:10.1167/tvst.13.4.19)

## Supplementary

**Supplementary Figure 1:** Two random examples for each of the four ordinal levels of quality, as graded by SK.
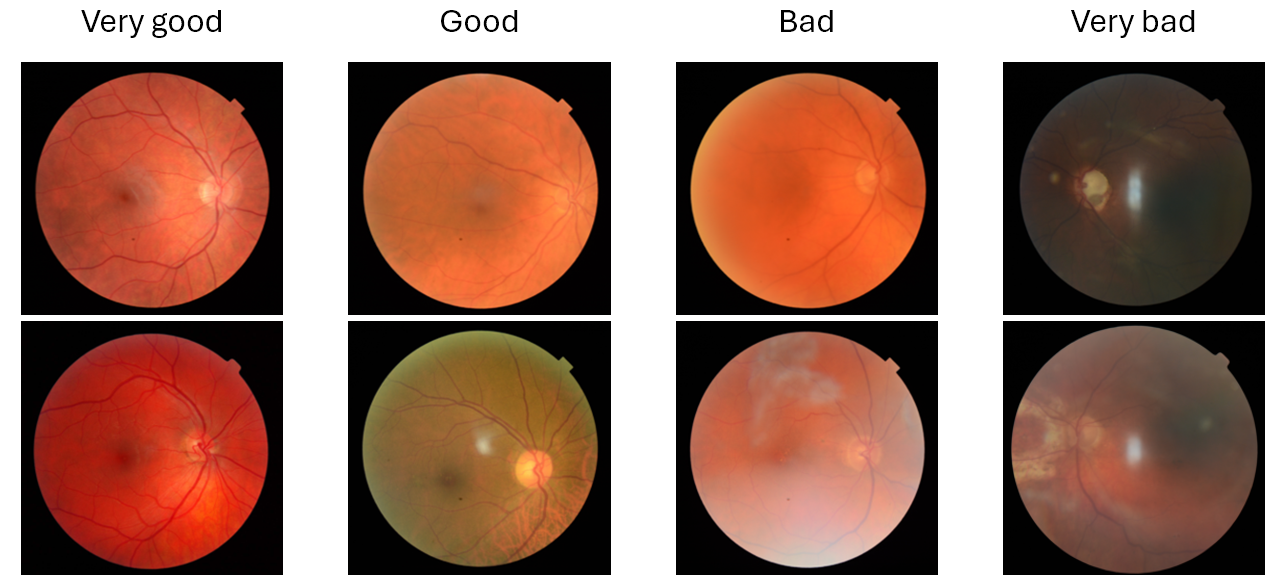

Supplement: Supplement 1 [file tvst-13-4-19_s001.docx]
